# Single‐cell mitochondrial lineage tracing: Opportunities and challenges

**DOI:** 10.1002/qub2.70018

**Published:** 2025-09-25

**Authors:** Siqi Li, Kun Wang, Xin Wang, Zheng Hu

**Affiliations:** ^1^ Department of Biomedical Engineering Southern University of Science and Technology Shenzhen Guangdong China; ^2^ State Key Laboratory of Quantitative Synthetic Biology Shenzhen Institute of Synthetic Biology Shenzhen Institutes of Advanced Technology Chinese Academy of Sciences Shenzhen Guangdong China; ^3^ Faculty of Synthetic Biology Shenzhen University of Advanced Technology Shenzhen Guangdong China; ^4^ School of Mathematical Sciences Xiamen University Xiamen Fujian China; ^5^ School of Basic Medical Sciences Chongqing Medical University Chongqing China

**Keywords:** lineage tracing, mtDNA variants, phylogenetic reconstruction, single‐cell genomics

## Abstract

Lineage tracing using endogenous mitochondrial DNA (mtDNA) variants holds great promise for reconstructing the lineage histories of individual cells, with broad applications in oncology, developmental biology, and regenerative medicine. Unlike synthetic DNA barcoding techniques, mitochondrial lineage tracing does not require genetic engineering of exogenous genetic markers, and thus is particularly suitable for human clinical samples. Various experimental and computational methods have been developed to profile mtDNA variants from single‐cell genomic, transcriptomic, and epigenomic sequencing data. Despite the technical advances, several challenges still limit the robustness of single‐cell mitochondrial lineage tracing, such as random genetic drift, genetic bottlenecks, informative variant identification, and low mtDNA coverage. In this review, we systematically examine current experimental and computational approaches for analyzing mtDNA variants in single cells and discuss current challenges and future technical developments aimed at enhancing the robustness and applicability of single‐cell mitochondrial lineage tracing.

## 1 SINGLE‐CELL MITOCHONDRIAL LINEAGE TRACING

Multicellular organisms are typically composed of diverse cell types, responsible for carrying out distinct biological processes that are essential to life. Unraveling the cell‐fate decision and regulatory mechanism underlying the heterogenous makeup of cell types is a longstanding goal of developmental biology [1] and cancer biology [2]. Lineage tracing is a powerful technique for tracing all progenies of a progenitor cell, thus exhibiting great potential for recording phylogenetic relationship in a broad spectrum of biological scenarios [3, 4, 5, 6]. The seminal work by John Sulston utilized microscopic observation to uncover the complete cell division history and cell fate transitions during the embryogenesis of *Caenorhabditis elegans*, displaying the power of lineage tracing in studying lineage dynamics [7]. However, its applications have been limited to transparent tissues or organisms due to its reliance on microscopy. Moreover, the low‐throughput of microscopic observation has further limited its use in more complex organisms, such as mammals [8].

Recent advances in genetic engineering and sequencing technologies collectively accelerated the development of novel lineage tracing methods, such as DNA barcode‐based techniques. These methods use, instead of vital dye or fluorescent protein, exogenous DNA sequences to label individual cells and these labeled cells, at a later time point, are sequenced to retrieve the integrated DNA barcode information and reconstruct the clonal relationship [9]. Additionally, emerging gene editing techniques, such as CRISPR/Cas9 and base editing, enable precise and efficient editing at desired genomic loci, opening a new avenue for high‐resolution lineage tracing [10, 11]. The integration of gene editing techniques into the DNA barcoding system allows the generation of mutations during cell division and provides a dynamic DNA barcoding system that is able to record high‐resolution phylogenetic relationships. These new methods have been applied to various biological scenarios and have generated high‐resolution phylogenetic trees, yielding novel insights into diverse biological processes. For instance, Lu et al. exploited a dynamic DNA barcoding system, substitution mutation‐aided lineage tracing, to interrogate the origin and evolutionary dynamics of intestinal tumorigenesis and revealed a polyclonal‐to‐monoclonal transition during the precancerous evolution of colorectal cancer, which challenged the classical monoclonal origin theory [12]. Despite the unprecedented phylogenetic resolution offered by these new methods, they lack the phenotypic readout of cells (e.g., cell type information). The incorporation of single‐cell omics techniques enables the simultaneous profiling of lineage identity and phenotypic state of single cells, allowing for a more systematic interrogation of cell fate determination [13].

Although these DNA barcoding‐based methods achieve high‐resolution and high‐throughput lineage tracing in diverse biological settings, their application in native human samples are limited due to their dependence on genetic engineering of synthetic DNA barcodes [14]. To cope with this, endogenous markers that naturally arise and accumulate in cells are used for tracing cell lineages. These endogenous markers include, but not limited to, single nucleotide variation [15, 16], copy number variation [17], microsatellites repeats [18], loss of heterozygosity [19], L1 retrotransposition elements [20], DNA methylation [21], and mitochondrial DNA (mtDNA) mutations [22, 23, 24, 25]. Nuclear SNVs have long been used for inferring lineage history in various contexts, but it remains challenging and costly to detect nuclear SNVs in single cells [26].

**FIGURE 1:**
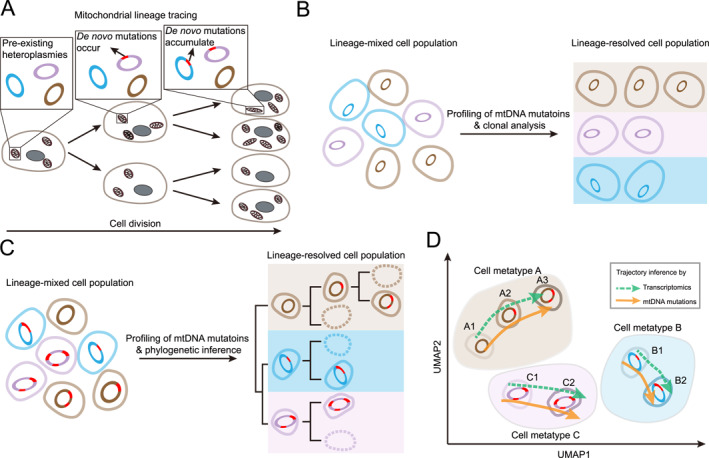
Schematic of single‐cell lineage tracing using endogenous mtDNA variants. (A) Schematic of principle of mitochondrial lineage tracing. Pre‐existing mtDNA mutations exist in cells and de novo mutations naturally arise and accumulate over cell divisions, thus becoming endogenous markers for lineage tracing. (B) Analysis of clonal relationships using mtDNA mutations. (C) Phylogenetic inference using mtDNA mutations. (D) Single‐cell transcriptomics coupled with mitochondrial lineage tracing provide phenotypic readout of single cells and provide faithful trajectory inference. mtDNA, mitochondrial DNA.

Mitochondrial DNA mutations are naturally generated at a higher rate, 10–100 times higher than nuclear genome [27]. Additionally, mtDNA is short in length (16,569 bp) and present in multiple copies in single cells, a feature that facilitates its detection [28]. Mitochondrial lineage tracing utilizes inheritable markers from mtDNA for tracing clonal populations and even single‐cell phylogenies (Figure 1A). Importantly, the availability of mtDNA mutations in a wide range of sequencing data, such as whole‐genome sequencing [29], transcriptomic [30, 31], and epigenomic data [23, 32], accelerates the application of mitochondrial lineage tracing, providing novel biological insights, especially in native human samples.

Indeed, mitochondrial lineage tracing has been applied to various biological contexts to study the lineage history [6, 32]. Naturally occurring mtDNA mutations can be seen as distinct natural DNA barcodes and cells tagged with the same mtDNA mutation should then form a clonal population (Figure 1B). Like nuclear genomic mutations, mtDNA mutations accumulate over time; therefore, these mtDNA mutations collectively enable phylogenetic inference (Figure 1C). Although mtDNA mutations have shown great promises in inferring phylogenetic relationships, lineage information alone only shows limited power in delineating complex biological processes. Emerging methods that couple single‐cell omics techniques with mitochondrial lineage tracing yield multimodal measurements of single cells and bridge lineage information with phenotypic readout, showing great promises in studying cell fate determination in complex systems [6, 22, 30, 32, 33] (Figure 1D).

## 2 GENOMIC APPROACHES FOR DETECTING mtDNA MUTATIONS IN SINGLE CELLS

Given the promises of single‐cell mitochondrial lineage tracing, the detection of mtDNA mutations in single cells has become increasingly important. Apart from the utilization of existing methods for the identification of mtDNA mutations, efforts have been made to develop novel single‐cell genomic methods for detecting mtDNA mutations preferentially along with multimodal phenotypic measurements. These methods can be classified into two categories: (1) direct mtDNA‐based methods that operate on mtDNA; (2) mitochondrial cDNA‐based methods that detect the mutations of transcribed products of mitochondrial genes, a proxy of mtDNA. In this section, we present an overview of representative single‐cell omics methods for the detection of mtDNA mutations spanning both categories, providing practical guidance for applying single‐cell mitochondrial lineage tracing.

### 2.1 Direct mtDNA‐based methods

#### 2.1.1 Single‐cell whole‐genome sequencing

Single‐cell whole‐genome sequencing (scWGS) profiles the complete genetic makeup of single cells, including both nuclear and mtDNA. The processes of scWGS include whole‐genome amplification, library preparation, sequencing, and computational analyses [34]. WGS data typically contains around 0.5% of mtDNA reads, although this proportion can vary depending on the copy number of mtDNA in each cell [23]. Because of the large number of read counts in WGS data by next‐generation sequencing, a high coverage of mitochondrial genome is typically observed in WGS data. For instance, a WGS dataset with around 1x coverage of nuclear genome typically contains a total of 10 million reads (PE150), of which approximately 50,000 are mtDNA‐derived reads, corresponding to around 900x coverage of mitochondrial genome. Therefore, WGS enables the detection of mtDNA variants at relatively low variant allele frequency (VAFs). However, due to the large size of the nuclear genome, the cost of scWGS is still high, resulting in a generally low throughput.

#### 2.1.2 scMito‐seq

Although scWGS can detect mtDNA variants in single cells, the high cost and low throughput limit its application in mtDNA‐based lineage tracing. Ludwig et al. described an approach for high‐throughput and unsupervised tracing of cellular clones and phenotypic states at single‐cell resolution in native human cells by mtDNA mutation detection [22]. They introduced the rolling circle amplification (RCA) technique for mtDNA amplification in individual cells and improved the genomic coverage and facilitated the detection of mtDNA variants [13]. scMito‐seq yields a higher mitochondrial genome coverage and a higher fidelity, which helps to reduce the cost to some extent [22, 35]. However, the amplification bias introduced by RCA may also lead to skewed mtDNA mutational profiling, thereby compromising the efficacy of mitochondrial lineage tracing.

#### 2.1.3 Single cell multiplexed probe targeted amplification sequencing

To achieve a deep profiling of mtDNA at the single‐cell level, the scSTAMP (single cell multiplexed probe targeted amplification sequencing) method was developed [36, 37]. This method utilizes 46 pairs of customized single‐stranded oligonucleotide probes to capture the entire human mtDNA genome via an extension‐ligation reaction, which largely increases the coverage of mtDNA. The technology has been validated in B lymphocytes and monocytes and has generated deep mtDNA mutation profiles (medium mtDNA coverage >100) for most cells, paving the way for accurate and sensitive detection of mtDNA mutations at single‐cell level [36]. However, scSTAMP is a well‐based method, so its throughput is still limited, especially compared with droplet‐based methods. More importantly, scSTAMP does not generate phenotypic measurements of single cells, hindering the decoding of cell states.

#### 2.1.4 mtscATAC‐seq

Mitochondrial genome is highly accessible due to the absence of histone protection, so sequencing reads of mtDNA make up a prominent proportion (around 8%) in bulk ATAC‐seq datasets [23]. Moreover, these mtDNA‐derived reads are uniformly distributed along the mitochondrial genome, achieving a near‐uniform coverage of mitochondrial genome [25]. The high content of mtDNA in ATAC‐seq highlighted the potential of ATAC‐seq for identifying mtDNA mutations. However, scATAC‐seq procedure, widely used in the field, such as 10X platform, involves cell lysis and nucleus isolation, thus losing a significant portion of mtDNA molecules. To recover more mtDNA, Lareau et al. established mtscATAC‐seq, a single‐cell mitochondrial assay for transposase‐accessible chromatin [25, 32]. The primary improvement of mtscATAC‐seq is to process whole cells to retain mtDNA by mild cell lysis or permeabilization. Additionally, mtscATAC‐seq simultaneously measures mtDNA heteroplasmy and chromatin accessibility in individual cells and therefore can be used to infer clonal relationships and cell state [23, 25, 32]. Collectively, mtscATAC‐seq is a powerful single‐cell mtDNA sequencing tool with high throughput and near‐uniform mtDNA genome coverage, allowing for high‐confidence variant identification and simultaneous inference of the cell state [32].

#### 2.1.5 ReDeem

ReDeem is a method that combines single‐cell regulatory multiomics with deep mitochondrial mutation profiling and is designed to achieve the simultaneous detection of mtDNA mutations, transcriptome, and chromatin accessibility at single‐cell resolution. ReDeem uses mtDNA‐specific probes to hybridize with Tn5‐tagmented mtDNA, which further enhances mtDNA enrichment and improves mtDNA coverage, thus enabling detection of low‐frequency heterozygous mutations. Additionally, ReDeem adopted a unique molecular identifier (UMI)‐based mtDNA variant calling method, which improves the precision of mutation calling. Collectively, ReDeem yields an approximately tenfold increase in mtDNA mutation detection. Meanwhile, ReDeem is capable of simultaneously capturing transcriptomic profiles, chromatin accessibility and lineage information of single cells, a feature enabling the integration of lineage information and functional phenotypes [6]. Indeed, ReDeem successfully revealed the clonal dynamics and functional diversity of human hematopoietic stem cells by integrating lineage information with multiomics data. Lastly, because ReDeem is based on a droplet platform, it achieves a high sequencing throughput and is suitable for large‐scale cohort studies.

### 2.2 Mitochondrial cDNA‐based methods

#### 2.2.1 Smart‐Seq2

Smart‐Seq2 is a well‐based single‐cell transcriptomic profiling technique that captures the entire transcribed region instead of 5′ ends or 3′ ends [38]. Because more than 90% of mitochondrial genome is transcribed, the full‐length RNA‐seq technique (e.g., Smart‐Seq2) that captures the entire sequence of a transcript can also be used to capture genetic variants [39]. Moreover, the transcription of mitochondrial genes is generally active to support the energy supply, resulting in a high content of mitochondrial transcripts within the full transcriptome. Therefore, Smart‐Seq2 provides both transcriptomic landscape and mtDNA mutation profiles, enabling integrative analyses of clonal relationships and cellular states [22, 40]. However, the high degree of inherent transcriptional error of the mitochondrial RNA polymerase and frequent RNA editing events may introduce inconsistency between mitochondrial genome and mitochondrial transcripts, further confounding the identification of mtDNA variants [41]. Besides, Smart‐Seq2 does not profile intergenic regions of mitochondrial genome, where mtDNA mutations could also arise. In addition, Smart‐Seq2 is a well‐based method and requires separate processing of each cell, so the throughput is typically limited and much lower than that of droplet microfluidics (e.g., 10X Genomics). Overall, Smart‐Seq2 shows great promises for single‐cell mitochondrial lineage tracing, but the high cost and relatively low throughput limit its application in reconstructing clonal relationships of complex human tissues.

#### 2.2.2 MutaSeq

Building on top of Smart‐Seq2, MutaSeq adopts specific primers for enriching genes of interest, such as mitochondrial genes, during the polymerase chain reaction (PCR) amplification step and carries out the simultaneous detection of gene expression and somatic variation in nuclear genome and mtDNA [40]. MutaSeq has shown significantly higher coverage of targeted region than conventional Smart‐Seq2, thereby displaying superior performance in the identification of mtDNA mutation identification. Moreover, the accuracy of MutaSeq has been benchmarked by a parallel deep whole exome sequencing, demonstrating the high accuracy of MutaSeq. More importantly, MutaSeq also provides comprehensive transcriptomic profiles of single cells, enabling the inference of cellular states. However, the use of a big number of primer pairs (e.g., >40) lead to a progressive decrease of library quality, limiting the scalability of MutaSeq [40]. Additionally, because MutaSeq is based on Smart‐Seq2, it also shares the same disadvantages with Smart‐Seq2, such as a relatively low throughput and a high error rate introduced by error‐prone cDNA synthesis. These technical characteristics of MutaSeq have limited its applications in diverse biological contexts.

#### 2.2.3 Mitochondrial alteration enrichment from single‐cell transcriptomes to establish relatedness

To scale up the throughput, MAESTER (mitochondrial alteration enrichment from single‐cell transcriptomes to establish relatedness), a droplet platform‐based single‐cell sequencing technology that captures mtDNA variants, was developed. MAESTER adopts the same strategy as MutaSeq and it uses a custom primer set targeting mitochondrial genes to amplify mitochondrial transcripts from labeled cDNA library, which ensures the coverage of mitochondrial genome. Miller et al. showed that MAESTER significantly improved mitochondrial transcript coverage by 50‐fold compared to scRNA‐seq data alone [24]. Importantly, this method can be integrated into multiple platforms, including 10X Genomics 3′ protocols, Seq‐Well S3 and Drop‐seq, potentially extending its application to diverse biological scenarios. In addition, because it is coupled with scRNA‐seq, MAESTER also provides cell state information in addition to lineage readout. However, the enrichment process of mitochondrial transcripts may also be biased due to the use of custom primers targeting different loci of mitochondrial genes, which may ultimately lead to an uneven coverage of mitochondrial genome.

A detailed comparison demonstrating features of single‐cell sequencing technologies for detecting mtDNA mutations is shown in Table 1.

**TABLE 1:** Comparisons between sequencing technologies for detecting mtDNA mutations in single cells.

Method	Principle	Coverage of mtDNA	Throughput	Multi‐omics integration
scWGS [34]	Single‐cell whole genome amplification (MALBAC/MDA) combined with high‐throughput sequencing	Low	Low	No
scMito‐seq [22]	mtDNA RCA combined with single‐cell sequencing	High	Low	No
scSTAMP [36, 37]	Targeted amplification of mtDNA using multiplex probes followed by single‐cell sequencing	High	Low	No
mtscATAC‐seq [25, 32]	Tn5 transposase preferentially cleaves open chromatin (including mtDNA), combining ATAC‐seq with mtDNA genotyping	Median	High	Epigenome
ReDeem [6]	Single‐cell multi‐omics (ATAC + RNA) combined with mtDNA enrichment	High	High	Transcriptome + epigenome
Smart‐Seq2 [39]	Full‐length transcriptomic profiling (predominantly capturing polyA mRNA)	High	Low	Transcriptome
MutaSeq [40]	Smart‐Seq2 combined with mitochondrial transcriptome enrichment	High	Low	Transcriptome
MAESTER [24]	Common 3′ single‐cell RNA‐sequencing protocols combined with mitochondrial transcriptome enrichment	High	High	Transcriptome

Abbreviations: MAESTER, mitochondrial alteration enrichment from single‐cell transcriptomes to establish relatedness; MDA, multiple displacement amplification; mtDNA, mitochondrial DNA; RCA, rolling circle amplification; scSTAMP, single cell multiplexed probe targeted amplification sequencing; scWGS, single‐cell whole‐genome sequencing.

## 3 CHALLENGES OF SINGLE‐CELL MITOCHONDRIAL LINEAGE TRACING

Although single‐cell mitochondrial lineage tracing has shown promises in diverse biological contexts, the unique features of mitochondrial biology have introduced uncertainties to this system. For example, mtDNA is present in multiple copies within cells and these copies are randomly allocated to daughter cells during cell division, resulting in drift of mutation frequencies. In addition, mitochondrial bottleneck events may cause more rapid and dramatic changes of mtDNA mutation frequencies. Lastly, the horizontal transfer of mitochondria between cells leads to unwanted alterations of mtDNA mutation profiles, which may interfere with phylogenetic reconstruction. In this section, we discuss how the performance of single‐cell mitochondrial lineage tracing may be influenced by genetic drift, mitochondrial bottleneck effect, and mitochondrial transfer.

### 3.1 Genetic drift

**FIGURE 2:**
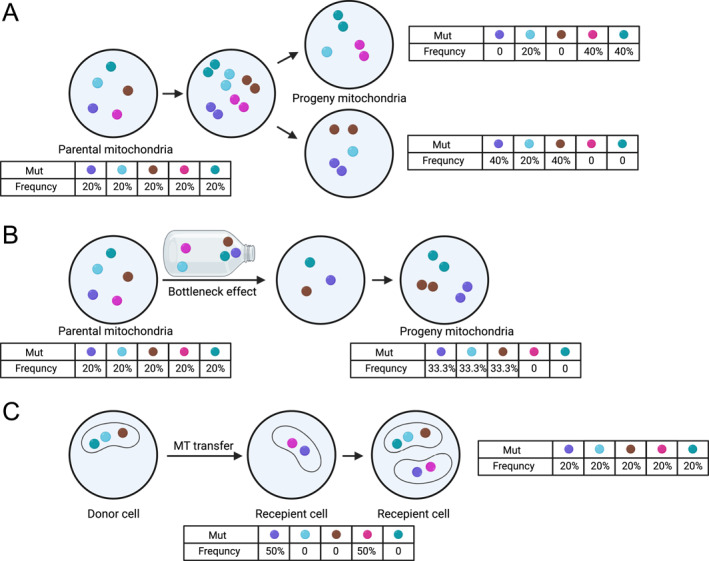
Schematic showing three potential confounding factors of single‐cell mitochondrial lineage tracing. (A) Genetic drift, introduced by the random allocation of mitochondria during cell division, leads to altered mtDNA mutation frequencies across generations. (B) The bottleneck effect results in a decreased content of mitochondria in the progeny, introducing drastic changes to mtDNA frequencies. (C) Mitochondria transfer from the donor cell to the recipient cell alters the mutation frequency in both the donor and the recipient cell. mtDNA, mitochondrial DNA.

Genetic drift refers to fluctuations in variant allele frequencies which is pronounced in small populations. In mtDNA lineage tracing, genetic drift is manifested as a random change in the proportion of mtDNA mutations among daughter cells due to the random allocation of mitochondria during cell division (Figure 2A) [42, 43, 44]. The unique biological features of mitochondria, such as the high copy number of mtDNA, the vegetative segregation that occurs during division and the relaxed replication of mtDNA, collectively lead to the susceptibility of mtDNA to genetic drift during division [45, 46, 47]. As a result of the genetic drift, the frequency of mitochondrial mutations may rise or fall rapidly in some clones within a short period of time while remaining relatively stable in other clones, confounding the reconstruction of genealogical relationships at the single‐cell level. Additionally, populational dynamics, such as clonal expansion, plays an important role in shaping the clonal structure of a given cell population. For example, clonal expansion often leads to an overrepresented subpopulation originating from the same ancestor; therefore, a cell population with prominent clonal expansions generally displays a low lineage complexity, representing an optimal setting for mitochondrial lineage tracing [30]. Taken together, genetic drift introduced by the complex mitochondrial biology and populational dynamics significantly reshapes the mtDNA mutational profiles, which introduces uncertainties to mitochondrial mutation‐based lineage tracing [29].

### 3.2 Bottleneck effect

Bottleneck effect refers to the phenomenon that during cell division the amount of mtDNA is significantly reduced, resulting in a lower content of mtDNA molecules in daughter cells (Figure 2B). This phenomenon is particularly evident during oocyte development and may lead to a rapid loss of mtDNA mutations in the progeny, significantly altering heteroplasmies of mtDNA mutations [48, 49]. More specifically, during a mitochondrial bottleneck, a few mtDNA copies carrying mutations may get stochastically selected and passed on to the progeny, which leads to a rapid increase of the frequency of selected mtDNA mutations. Besides these retained mitochondria, the rest of the mitochondria that also carry mtDNA mutations would likely disappear, leading to the decrease of heteroplasmic mtDNA mutations or even the loss of low‐frequency mutations. Therefore, the mitochondrial bottleneck may create some high‐frequency or even fixed (VAF = 100%) mutations and eliminate some low‐frequency mutations, resulting in a distortion of mtDNA mutational landscape. We previously reported that mtDNA mutations with high‐frequency and low variance are more likely to be informative for clonal inference [30]; therefore, a mitochondrial bottleneck that accelerates the generation of high‐frequency mutations could be beneficial. However, the elimination of mutations could lead to a loss of phylogenetic signal which could have been carried by those eliminated mutations. Overall, the mitochondrial bottleneck effect may introduce rapid and dramatic change of the mutation profiles and the distorted mutation profiles may disrupt the phylogenetic inference, thus complicating the application of mitochondrial lineage tracing.

### 3.3 Mitochondrial transfer

Recent studies have revealed that mitochondria can be obtained by either inheriting from parental cells though cell division or horizontal transfer from other donor cells. Mitochondrial transfer is usually mediated by tunneling nanotubes (Figure 2C), but it can also be achieved by other means, such as cell fusion, intercellular connections (e.g., gap junctions), exosomes (EVs), and direct cell‐to‐cell contact [50, 51]. This phenomenon has been observed in a variety of cell types, especially between cancer cells and immune cells [51]. The bidirectional transfer of mitochondria may simultaneously enhance the energy metabolism of cancer cells [51, 52] and suppress the antitumoral function of immune cells [53], thus playing a role in tumor progression. In the context of mitochondrial lineage tracing, the transferred mitochondria introduce new mutations to the recipient cell. The separation of transferred mitochondria from the locally inherited mitochondria population is extremely challenging or impossible with current methods, so the mutations carried by those transferred mitochondria from donor cells would be falsely treated as part of the recipient cell. Furthermore, these mutations gained by mitochondrial transfer may falsely label the donor and the recipient as closely related cells, even though they may be quite distant from each other on the actual phylogenetic tree. Therefore, mutations introduced through mitochondrial transfer are likely to produce misleading phylogenetic signals, ultimately leading to inaccurate genealogical inference. Taken together, mitochondrial transfer represents a confounding factor for applying mitochondrial lineage tracing and the frequency of mitochondrial transfer should be carefully estimated and taken into consideration when applying mitochondrial lineage tracing.

### 3.4 Identification of informative mtDNA mutations

**FIGURE 3:**
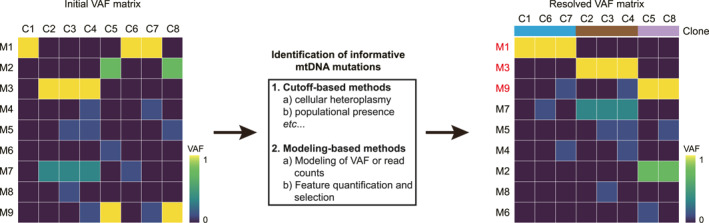
Identification of informative mtDNA mutations for analyzing clonal relationships. The mtDNA mutation profiles (VAF matrix) are processed with different pipelines/algorithms to resolve the clonal relationship of the cell population. Mutations highlighted in red are putative informative mtDNA mutations for clonal analysis. Rows: mutations, columns: cells. mtDNA, mitochondrial DNA; VAF, variant allele frequency.

Given the complex nature of mitochondrial biology, identifying mtDNA mutations that are informative for clonal tracking is not as straightforward as nuclear SNV‐based lineage tracing methods. Along with the development of single‐cell mitochondrial lineage tracing methods, computational tools that aim to identify informative mtDNA mutations have also been developed. Initially, empirical cutoffs for heteroplasmies and populational presence (i.e., the fraction of cells carrying a specific mutation) of mtDNA mutations were set to screen for informative mtDNA mutations (Figure 3). For example, Ludwig et al. selected mtDNA mutations that were present at a minimum heteroplasmy of 5% and in at least 80% of cells within a predefined clonal cell population and identified informative mtDNA mutations that successfully recovered the ground‐truth clonal relationships [22]. These sorts of methods are easy to apply and do not require additional computational tools, and thus they have been widely used in the field [4, 22, 24, 30, 54]. However, these methods based on empirical cutoffs fail to identify informative mtDNA mutations with low heteroplasmies or populational presence.

In light of this, new methods that adopt more sophisticated computational modeling for feature selection have achieved a better performance on the identification of informative mtDNA mutations (Figure 3). For example, MQuad first fits reference and alternative read counts of a specific variant to a binomial distribution with two distinct assumptions: (1) without clonal structure (i.e., all cells share the mutation) and (2) with clonal structure. Then, the Bayesian information criterion (BIC) scores of both models are calculated, and the difference between two models (ΔBIC) are used for quantifying the informativeness of individual mtDNA mutations [31]. MitoTracer, unlike MQuad, assumes that the herteroplasmy level of a mutation across all cells follow a Gaussian mixture distribution and uses Dirichlet process (DP) prior to determine the Gaussian peaks, indicative of number of clones. Subsequently, the peak positions of the top two distributions assigned by DP are compared and the distance is then used as a metric to estimate the lineage signal of the selected mtDNA mutations [55]. Additionally, another computational framework, LINEAGE, focus on both mutation type diversity and dynamic pattern separation, which helps avoid bias and improve accuracy. This framework first identifies highly variable sites, groups these highly variable into subspaces by variant dynamics, and calculates the cross‐entropy among these subspaces. Those subspaces with lower cross‐entropy are more likely to reflect the real clonal structure and are used for the following consensus clustering. Finally, mtDNA mutations characterizing each cluster are selected as informative mtDNA mutations for clonal tracking. LINEAGE has been applied to a genetically barcoded cell population and successfully resolved the clonal structure, showing its great potential in the identification of informative variants [56].

### 3.5 Phylogenetic inference using mtDNA mutations

Given the inherently high mutation rate of mtDNA, somatic mutations provide robust markers for phylogenetic inference at single‐cell resolution. Conventional methods typically infer phylogenetic trees using maximum‐likelihood [57], maximum‐parsimony [58], neighbor‐joining [59, 60] approaches, or Bayesian frameworks [61] under suitably chosen substitution models, analogous to those applied in nuclear DNA analyses. However, distinctive features of mtDNA preclude the direct application of methods developed for nuclear genomes [30].

**FIGURE 4:**
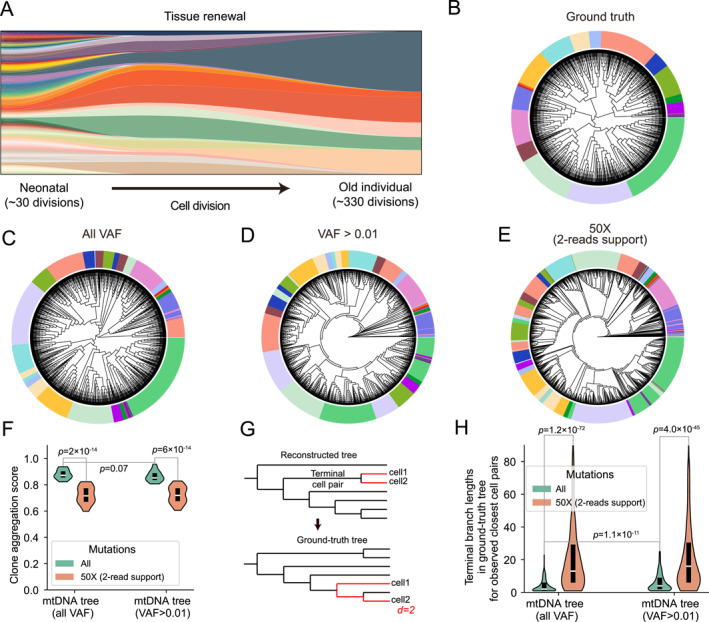
Computational simulation of mitochondrial lineage tracing in single cells. (A) Schematic of tissue renewal stage. Cell population remains constant while the number of lineages decreases as cell division. (B) Simulated ground‐truth single‐cell phylogenetic tree in neonatal stage. The colors indicate clones defined by 20 clones. (C) Reconstructed phylogenetic trees using all mtDNA variants. (D) Reconstructed phylogenetic tree using mtDNA variants with VAF > 0.01. (E) Reconstructed phylogenetic tree using mtDNA variants with 2‐reads support and 50X sequencing coverage. (F) Violin plot showing CAS across different VAF cutoffs. Colors indicate sequencing conditions: green for no sequencing and orange for 50X sequencing with 2‐read support for identification of mtDNA variants. (G) Schematic illustrating the calculation of terminal branch length. The closest cell pairs are first identified in the reconstructed tree (top), and then terminal branch lengths are measured in the ground‐truth tree (bottom). (H) Violin plot showing terminal branch lengths in the ground‐truth tree for the closest cell pairs observed in the reconstructed tree. Colors represent sequencing conditions: green for no sequencing and orange for 50X sequencing with 2‐read support for identification of mtDNA variants. CAS, clone aggregation scores; mtDNA, mitochondrial DNA; VAF, variant allele frequency.

To illustrate the impact of these mtDNA dynamics on phylogenetic reconstruction, we conducted in silico simulations of cell division that incorporated both mtDNA replication and random segregation (Figure 4A). Phylogenetic trees were constructed from the resulting mtDNA mutation profiles using a neighbor‐joining algorithm. To rigorously assess the effectiveness of mtDNA variant‐based lineage reconstruction, we first evaluated the accuracy of major lineage clustering. Specifically, cells at the sampling time point were divided into 20 clones with similar clone sizes according to their phylogenetic distance (Figure 4B). The simulated data were subsequently processed under three conditions:

1. All VAF—all detected mtDNA variants,
2. VAF > 0.01—only variants with a mutation frequency >1%,
3. Simulated 50X sequencing with 2‐read support—variants supported by at least two reads.

For each dataset, reconstructed lineage trees were annotated with genuine clone assignment and a tighter clustering of cells sharing the same color was indicative of more accurate clonal reconstruction. Our analysis of the late phase of development revealed that low‐VAF mutations play a significant role in lineage reconstruction. When all mutations, regardless of VAF, were used, the reconstructed lineage tree showed a good clustering of cells within the same clone (Figure 4C). Additionally, when mutations with low VAFs (<0.01) were excluded, the accuracy of clone assignment only marginally decreased while the topology of the reconstructed tree changed significantly (Figure 4D). Furthermore, when the sequencing process was introduced, the accuracy of the reconstructed lineage tree further declined indicated by the altered topology and intermixed cell clusters (Figure 4E). Subsequently, we introduced a metric, clone aggregation score (CAS) [30], to quantitatively assess the accuracy of clone assignment using mtDNA mutations (Supporting Information S1). We found that the simulated sequencing process significantly decreased the accuracy of mtDNA mutation‐based lineage tracing. Additionally, the integration of low‐VAF mtDNA mutations could not significantly increase the accuracy of mitochondrial lineage tracing at clone level (Figure 4F). To better quantify the accuracy of reconstructed phylogenetic trees at a finer resolution, we traced the phylogenetic relationship of closest cell pairs observed in reconstructed phylogenetic trees and calculated their distance in the ground‐truth trees (Figure 4G, Supporting Information S1). A small value of this distance is indicative of high accuracy of phylogenetic reconstruction. Similar to previous results (Figure 4F), the simulated sequencing process led to a significant decrease of lineage tracing accuracy in both groups (Figure 4H). More importantly, the exclusion of low‐VAF mtDNA mutations prominently increased the distance (Figure 4H), indicating that low‐VAF mtDNA mutations may preserve critical information for fine‐scale phylogenetic lineage reconstruction.

## 4 FUTURE PERSPECTIVES

### 4.1 Technology development

Given the unprecedented power of low‐frequency mutations in lineage reconstruction (Figure 4), the development of single‐cell sequencing methods with improved coverage of mtDNA should be warranted. Currently, single‐cell mitochondrial lineage tracing methods heavily depend on PCR for the amplification of mtDNA as previously described (Table 1). Therefore, a systematic optimization of primers and reaction conditions should be performed to minimize the amplification bias and maximize amplicon yield. Moreover, the incorporation of UMI into the amplification process may help to correct for the amplification bias. Additionally, targeted enrichment techniques can also be combined with PCR amplification to further increase the yield of mtDNA amplicon, thereby improving mtDNA coverage. Moreover, for methods that are based on mtDNA (e.g., mtscATAC‐seq), it is critical to retain mitochondria from the cytoplasm; therefore, the optimization of experimental protocols, such as mild cell lysis, should be conducted to avoid the loss of mitochondria. Lastly, in addition to small‐size variations, the identification of large‐size variations may also be valuable for lineage tracing. Novel sequencing methods, such as Oxford nanopore technology and PacBio (Pacific Biosciences), generate long reads and are capable of identifying various types of genomic variations, potentially providing more variants of distinct types for lineage reconstruction [62].

The combination of lineage relationship and phenotypic state provides unique opportunities for understanding cell fate determinations and transitions. The incorporation of transcriptomic profiling enables the annotation of cell types and the identification of gene expression programs that are essential for cell fate determination. In addition, the further incorporation of chromatin accessibility provides additional regulatory insights, enabling a more systematic interrogation of cell fate determination. Future developments should incorporate diverse modalities of phenotypic information, such as DNA methylation and chromatin conformation, into mitochondrial lineage tracing to better understand the combinatory effect of lineage identity and phenotypic state on cell fate determination.

Spatial transcriptomic profiling has emerged as a promising technique for studying cell–cell interactions in the context of development and cancer [63]. The combination of spatial transcriptomics and lineage tracing can provide novel insights into how the interplay between genetic makeup of cancer cells (lineage) and external environmental cues shape the tumor progression and has shown great promises in delineating spatiotemporal dynamics during tumor evolution [64]. Currently, these methods are only available in nonhuman models. The incorporation of spatial transcriptomics into mitochondrial lineage tracing will extend the application of spatially resolved lineage tracing to native human samples. In addition, spatial information may provide instructions for mtDNA‐based lineage reconstruction. For example, the same mtDNA variant could arise in independent lineages due to the limited space for mutations, and spatial information may be supplemented to correct false lineage history inferred from this sort of mtDNA variants. Overall, future work aiming to (1) increase the coverage of mitochondrial genome, (2) provide multimodal phenotypic readout, and (3) incorporate spatial transcriptomics should be prioritized to accelerate the application of single‐cell mitochondrial lineage tracing.

### 4.2 Computational analysis

The field of single‐cell mitochondrial lineage tracing is poised for transformative growth as technological innovations push the limits of data resolution and analytical precision. Two particularly promising directions are the utilization of VAF information for precise phylogenetic inference and the integration of mtDNA phylogenies with transcriptome data for dynamic evolution analysis.

Although VAF has become a gap between mtDNA and nuclear DNA, it has also provided additional information for mtDNA lineage inference. Future methodological advancements will enhance our ability to extract reliable VAF signals from single‐cell datasets despite inherent technical challenges such as sequencing noise, dropout events, and low‐frequency mutations. Robust computational frameworks integrating statistical models and machine learning are expected to filter out platform‐specific biases and artifacts, thus enabling high‐resolution phylogenetic reconstructions. By accurately mapping VAF distributions, one would be able to elucidate mutation propagation, identify subtle lineage bifurcations, and ultimately resolve the timing and frequency of key cellular events.

Moreover, studies that integrate lineage information with transcriptomic data for single‐cell developmental inference are becoming increasingly important and have achieved significant breakthroughs [57, 65, 66, 67, 68, 69]. Integrating mtDNA phylogeny with transcriptomic data represents a frontier where static lineage information meets dynamic cellular function. The transcriptome offers a snapshot of gene expression that reflects a cell’s state, whereas mtDNA mutations provide a record of lineage relationships. Future studies are envisioned to leverage time‐resolved multi‐modal single‐cell data to trace transcriptional reprogramming during cell fate transition. Future efforts into the improvement of experimental protocols and analysis framework will expand the application of single‐cell mitochondrial lineage tracing and will yield novel mechanistic insights into cell fate transitions in somatic human tissues.

## AUTHOR CONTRIBUTIONS

**Siqi Li**: Formal analysis; investigation; writing—original draft; writing—review and editing. **Kun Wang**: Formal analysis; investigation; methodology; writing—original draft. **Xin Wang**: Conceptualization; formal analysis; investigation; methodology; writing—original draft; writing—review and editing. **Zheng Hu**: Conceptualization; supervision; writing—review and editing.

## CONFLICT OF INTEREST STATEMENT

The authors declare no conflicts of interest.

## ETHICS STATEMENT

This review article does not contain any human or animal studies conducted by any of the authors.

## Supporting information

Supporting Information S1

## Data Availability

Code for performing simulation was deposited at GitHub website (kunwang34/mtDNA_simulation). No sequencing data were generated or analyzed in this study.
