# Supplementary material for: Single‐cell mitochondrial lineage tracing: Opportunities and challenges
Source: Quant Biol. 2025 Sep 25;14(1):e70018. doi: 10.1002/qub2.70018 (PMC12806035; doi:10.1002/qub2.70018)
Supplement: Supplementary file 1 — Supporting Information S1 [file QUB2-14-e70018-s001.docx]

**Supplementary file 1**

The simulation of mitochondrial lineage tracing data is divided into three parts, simulation of stem cells growth, simulation of mitochondrial genome proliferation along with cell division and simulation of mtDNA variants sequencing data.

*Simulation of stem cells growth*

As our previous published study[1], we first simulate the stem cells growth using Gillespie algorithm[2]. By conceptualizing cell division as components of continuous-time Markov process, we designate the reaction rate of cell division as $p(t)$ given as follows:

$$p\left( t \right)=r\left( 1-\frac{1}{1+e^{-k\left( t-t_{0} \right)}} \right),$$

Where the three parameters $r,k$ and $t_{0}$ jointly determine the change of cell growth rate with time.

We then simulate the cell population growth with given division rate from 1 initial cell until the population size reached 20,000 cells. 1,000 cells were randomly sampled to obtain their division history.

*Simulation of mitochondrial genome proliferation over cell divisions*

To model the mitochondrial lineage tracing within a specified phylogenetic tree, an initial population of mitochondrial DNA was allocated to the progenitor cell. The proliferation dynamics of mitochondrial DNA were simulated using the Gillespie stochastic simulation algorithm, with division and death rates at 1 and 0.1, respectively. This simulation was terminated when the mitochondrial genome count reached 500 units. During the initial phase of mitochondrial genome generation, the mutation rate per division was set to follow a Poisson distribution with an expectation of 0.1 mutations per division.

After establishing the initial population of mitochondrial DNA, the model was extended to simulate the accumulation of *de novo* mutations within the mitochondrial genome as cellular division occurred and lineages diverged. When the cell divides, the replicated mitochondrial DNA are segregated to the two daughter cells, which follows binomial distribution. It has been estimated that per-mitosis mutation rate for mitochondrial genome is 10-100 higher than nuclear genome per site. Assuming the somatic mutation rate of nuclear genome as ~10^-9^ per mitosis per site, 100-fold increase in mutation rate for mitochondrial genome and 500 mitochondrial copies, each cell acquires ${10}^{-9}\times16,569\times100\times500\approx0.8$ mitochondrial mutations per mitosis on average. Hence, we set the number of mitochondrial mutations generated within the cell after each division to follow a Poisson distribution within an expectation of 0.8 mutations per division.

*Simulation of tissue renewal process*

To simulate tissue renewal, we modeled the update process for each cell lineage based on the simulations conducted during the development stage. For each cell lineage, we simulated cell division and the replication and allocation of mtDNA in the same manner as in the development stage. This approach resulted in two daughter cells, each containing approximately 500 mtDNA with new mutations. Next, we considered the renewal process. To maintain a roughly constant population of tissue cells, we used a Bernoulli distribution with $p=0.5$ to determine the proportion of progeny retained within the sampled tissue. This process was repeated for 100 times.

*Simulation of mtDNA variants sequencing data*

We first sampled the sequencing depth. Previous studies have shown that sequencing depth typically follows a negative binomial distribution. Here, we used a negative binomial distribution with an expected value of $\mu=50$ and the number of successes $n=2.5$ for sampling

$$Depth \sim nbinom\left( p=\frac{n}{n+\mu}, n=2.5 \right).$$

For the true VAF ($f$), we sampled its sequencing value using a binomial distribution to obtain the read count

$$readcount\sim Binom\left( p=f,n=Depth \right).$$

**Calculation of clone aggregation score**

As previously described[3], to quantify the accuracy of reconstructing major lineages from mtDNA mutations, we define the Clone Aggregation Score (CAS). First, we obtain the lineage information of cells from the ground truth tree. For the reconstructed tree $\mathcal{T}$, we define the clone switch value $s$. As we traverse all leaf nodes of the cells from left to right, $s$ increments by 1 whenever there is a lineage change in the ground truth tree. Clearly, the range of $s$ is between the number of lineages ($s_{min}$) and the number of cells ($s_{max}$). Therefore, we normalize $s$ to a range of 0 to 1 and take the logarithm to linearize it, resulting in the CAS:

$$CAS=1-\log\left( \frac{s_{min}}{s} \right)/\log\left( \frac{s_{min}}{s_{max}} \right).$$

**Calculation of terminal branch lengths in ground-truth tree for observed closest cell pairs**

To assess the accuracy of mtDNA variants in fine-scale lineage reconstruction, we first randomly select a cell from the reconstructed tree and identify its nearest neighbor through their most recent common ancestor node. These two cells are recorded as a cell pair. We then locate these two cells in the ground truth tree and calculate their distance in the lineage tree, which is half the sum of the branch lengths to their nearest common ancestor. This distance represents the terminal branch lengths in the ground-truth tree for observed closest cell pairs. This process is repeated 500 times for each reconstructed tree.

**Reference**

1. Friedman PA: **PTH revisited.** *Kidney Int Suppl* 2004**:**S13-19.

2. Azizova OA, Islomov AI, Roshchupkin DI, Predvoditelev DA, Remizov AN: **[Free radicals formed during UV-irradiation of biological membrane lipids].** *Biofizika* 1979, **24:**396-402.

3. Wang X, Wang K, Zhang W, Tang Z, Zhang H, Cheng Y, Zhou D, Zhang C, Zhong WZ, Ma Q, et al: **Clonal expansion dictates the efficacy of mitochondrial lineage tracing in single cells.** *Genome Biol* 2025, **26:**70.
